# Stigma about palliative care: origins and solutions

**DOI:** 10.3332/ecancer.2022.1377

**Published:** 2022-04-28

**Authors:** Jacqueline Alcalde, Camilla Zimmermann

**Affiliations:** 1 Department of Supportive Care, Princess Margaret Cancer Centre, Toronto M5G 2M9, Canada; 2 Divisions of Palliative Medicine and Medical Oncology, Department of Medicine, University of Toronto, Toronto M5G 2C1, Canada; 3 Division of Palliative Care, University Health Network, Toronto M5G 2C1, Canada; 4 Department of Psychiatry, University of Toronto, Toronto M5G 2C1, Canada

**Keywords:** palliative care, cancer, health policy, health education, public health, social stigma

## Abstract

Despite high-level evidence demonstrating the benefits of integrating palliative care early in the trajectory of advanced cancer, there remains a stigma surrounding this important discipline. This stigma is rooted in the origins of palliative care as care for the dying and is propagated by misinformation and late referrals to palliative care services. Current official definitions of palliative care emphasise the importance of early identification and treatment of symptoms and provision of care concurrently with treatments aimed at improving survival. However, this model of palliative care is neither widely known by patients and their caregivers nor consistently practiced. Herein, we describe changes that are necessary at the levels of practice, policy and public education to shift the *status quo*. Change requires palliative care teams that are staffed, trained and resourced to accommodate early referrals; education for referring physicians to provide high-quality primary palliative care, as well as timely referral to specialists; and a public health strategy for timely palliative care that educates and engages policymakers, stakeholders and the public. The hospice movement was directed at improving care for the dying; continued expansion of this movement is necessary so that all patients with advanced cancer may benefit from its principles throughout the course of illness.

## Introduction

Receiving a diagnosis of cancer is itself a fearful experience, but the introduction of ‘palliative care’ as a treatment option is perhaps equally traumatic. ‘Palliative care’ is perceived by patients and their caregivers – and often by healthcare providers – as meaning cessation of treatment, lack of hope and imminent death [1]. We argue that this association is rooted in the or﻿igins of palliative care in the hospice movement and is propagated by the continued practice of palliative care as end-of-life care, despite evidence that its early integration into cancer care improves quality of life and perhaps also survival.

## Origins of palliative care

Palliative care originated in the modern hospice movement in the 1960s. In 1967, Dame Cicely Saunders opened St. Christopher’s Hospice in London, where she developed an interdisciplinary model of care for patients approaching the end of life [2]. Saunders challenged the then-prevailing view that lack of an available cure constituted a failure on the part of the physician and considerably improved the standard of end-of-life care by introducing a multidisciplinary approach. Her philosophy drew the attention of clinicians worldwide, among them Dr Balfour Mount, a Canadian urologist, was interested in improving end-of-life care. Inspired by his visit to St. Christopher’s in 1973, Dr Mount founded a hospice-style inpatient unit at the Royal Victoria Hospital in Montreal, Canada [3]. As the word ‘hospice’ had a negative connotation in French, he introduced the term ‘palliative care’, which is based on the Latin ‘palliare’ meaning ‘to cloak’ [3, 4].

Palliative care was formally defined by the World Health Organisation (WHO) [5], as the ‘active total care of patients whose disease is not responsive to curative treatment with the goal of achieving best possible quality of life’. However, it soon became clear that this model of care was relevant throughout the course of a serious illness. The WHO [6] redefined palliative care as ‘an approach that improves the quality of life of patients and their families facing problems associated with life-threatening illness through the prevention and relief of suffering by means of early identification and impeccable assessment and treatment of pain and other problems, physical, psychosocial and spiritual’. However, the original definition based on end-of-life care has held fast and continues to represent the most commonly practiced model of palliative care [7].

## Evidence for the benefit of early palliative care

Although the 2002 WHO definition emphasised early intervention to improve quality of life, definitive evidence for this model of care did not emerge until almost a decade later. In 2010, a randomised controlled trial demonstrated improved quality of life and mood for patients with newly diagnosed advanced non-small cell lung cancer who received early specialised palliative care, compared with those who received standard care [8]. In 2014, a trial involving patients with advanced lung, gastrointestinal, genitourinary, breast and gynaecological cancers demonstrated improvement in quality of life, symptom control and satisfaction with care for patients randomised to early involvement of specialised outpatient palliative care, compared to standard care [9]. Further randomised trials in the United States and Europe reproduced these benefits of early specialised palliative care for patients with cancer; demonstrated benefits of improved mood and satisfaction with care for their caregivers; and showed a significant reduction in aggressive interventions at the end of life [10–13].

Thus, in the last two decades, strong evidence has demonstrated that involving specialised palliative care early in the trajectory of advanced cancer improves multiple outcomes among patients with advanced cancer and their caregivers. Consequently, multiple organisations now recommend early palliative care as a standard of care in advanced cancer; these include the American Society of Clinical Oncology and the European Society of Medical Oncology [14–16].

## Patient, caregiver and public perceptions of palliative care

Despite the evidence for the benefit of early palliative care and the endorsement of this model by oncology societies, palliative care carries negative connotations for patients, caregivers and the public [1, 17–20]. In a qualitative study conducted in a Canadian cancer centre, patients and caregivers in the control and intervention groups were interviewed after completion of a 4-month trial of early palliative care versus routine oncology care [1]. In both trial arms, participants stated that before beginning the trial, they associated palliative care with death, hopelessness, dependency and end-of-life care. Similar perceptions were reported in another qualitative study, in which patients were recruited from cancer services at a metropolitan hospital in Australia: patients and caregivers associated the term palliative care with a system of diminished care, diminished possibilities and diminished choice [17].

The Canadian qualitative study described above compared opinions of participants in the control and intervention arms before and after the trial [1]. While participants in the control group did not report a change in their opinions, those who received early specialised palliative care developed a more comprehensive concept of palliative care as ‘ongoing care’ that improved their ‘quality of living’. Despite this positive shift, the intervention group participants felt that the term palliative care carried a stigma and strongly suggested that palliative care should be reframed and better explained by healthcare professionals.

Public surveys have also demonstrated a lack of knowledge about palliative care [18–21]. Cross-sectional surveys from a wide range of countries have demonstrated that the public associates palliative care with end-of-life care [18, 19], and a recent Canadian survey revealed a gap in perceived and actual knowledge of palliative care [20]. Of 1,518 survey participants, 45% had a high self-rated knowledge of palliative care, yet less than half of these were aware of important elements of the WHO palliative care definition. Moreover, those who believed they had high knowledge of palliative care were less likely to believe palliative care offered hope than those who believed they had low knowledge; conversely, those with greater actual awareness of the WHO palliative care definition were more likely to believe palliative care offers hope. These surveys underline the importance of public education to decrease stigma about palliative care.

## Oncologists’ referral practices and perceptions of palliative care

Patients, caregivers and the public have identified experiences within the healthcare system as the main source of their opinions about palliative care [1, 18, 20]. Oncologists tend to refer patients late in the disease course when symptoms are poorly controlled and systemic treatments have been exhausted [7, 21]. Although cancer centres in North America have reported earlier referrals in recent years [22, 23], a similar pattern of earlier referral has not yet been observed in Europe [24].

Physicians’ referral practices reflect their attitudes towards palliative care. In surveys and qualitative studies, oncologists expressed ambivalence about the early integration of palliative care [25]. Some physicians associated palliative care with terminal care or with discontinuation of cancer treatment [26–28] or felt that the term palliative care could upset patients or caregivers [29, 30]. Others believed that palliative care specialists could interfere with the cancer care plan by introducing different treatment goals or alternate options for care [25, 27].

Renaming palliative care has been proposed as a solution to late referral. Indeed, surveys have shown that approximately 20%–30% of oncologists would refer patients earlier to outpatient palliative care if its name was changed to ‘supportive care’ [20, 29]. However, in a cross-sectional study surveying palliative care specialists in Canada, only 20% agreed that the specialty of palliative care should be renamed [31]. Of note, while 90% of these palliative care physicians supported early palliative care referral and had referral criteria in place to facilitate this practice, only 20% received referrals earlier than 6 months before patient death.

## Potential solutions

Potential solutions to reduce stigma and increase integration of palliative care must involve a multipronged approach at the levels of individual patients and clinicians, institutions and society. Change must begin with the palliative care team itself: palliative care clinicians generally welcome early consultations but their teams must be sufficiently staffed, trained and resourced to accommodate early referrals [31, 32]. Ideally, a screening mechanism should also be in place so that early palliative care is prioritised for those who need it most [33]. Although there are multiple practice models that integrate palliative care, outpatient clinics represent the main setting for patients to be seen early in the disease trajectory [34]. Other important components of models to improve early palliative care integration are an interdisciplinary team with effective communication and collaboration among care providers, the capacity to respond quickly to patient needs and skill in addressing problems that arise early in the disease course (e.g., complications of cancer treatment and psychological distress) [24, 34, 35]. At the institutional and policy level, teams require sufficient funding to be able to deliver palliative care to patients with a wide range of prognoses [36].

Once a model is in place that can accommodate early palliative care, referring physicians need to feel comfortable explaining and enacting this collaborative model, rather than a ‘hand-off’ model at the end of life. In this regard, training of oncologists and oncology nurses regarding palliative care referral is essential to dispel myths and misconceptions about palliative care [37–39]. Lectures and clinical rotations in palliative care should be integrated as essential components in the curricula of medicine and nursing schools [40]. Indeed, it has been shown that oncologists who have completed a rotation in palliative care tend to refer patients earlier [21]. On the other hand, palliative care teams cannot and should not provide all palliative care, and it is important for oncologists and primary care providers to have a basic knowledge of palliative care. For clinicians in practice, there are opportunities to pursue continuing medical education as well as formal linkages and mentoring opportunities with tertiary palliative care teams to build and maintain skills in the providing high-quality primary palliative care [16].

In addition to education for healthcare providers, public education is also essential so that the patients and caregivers are aware of the relevance and importance of early, integrated palliative care. Education at a public health level would be enhanced by engaging national leaders, stakeholders and society as stakeholders to develop and enact a public health strategy on palliative care [40, 41]. Potential solutions to inform the public include media campaigns similar to the ‘chain of survival’ for first aid [42] and didactic palliative care resources directed to patients and caregivers to enhance their knowledge and access to palliative care [43, 44]. These resources could be shared in the form of online materials, short videos or printed pamphlets [45].

## Conclusion

Despite strong evidence for the benefit of early palliative care, patients and their caregivers continue to fear palliative care referral and associate it with cessation of treatment and imminent death. The reason for the *status quo* is circular: referrals are made late due to the misperception that palliative care is end-of-life care, and palliative care remains synonymous with end-of-life care due to late referrals. The way out of this circle is to change the policy and practice at multiple levels, and to provide broad-based education about the expanded meaning of palliative care. Only a society that is aware of the benefits of early palliative care can demand that resources be allocated to integrate and prioritise this essential healthcare service.

## Conflicts of interest

The authors declare no conflicts of interest.

## Funding

Dr Zimmermann is supported by the Harold and Shirley Lederman Chair in Psychosocial Oncology and Palliative Care, held jointly at the Princess Margaret Cancer Centre, University Health Network, and the University of Toronto.
